# Prognostic factor analysis for breast cancer using gene expression profiles

**DOI:** 10.1186/s12911-016-0292-5

**Published:** 2016-07-18

**Authors:** Soobok Joe, Hojung Nam

**Affiliations:** School of Electrical Engineering and Computer Science, Gwangju Institute of Science and Technology, 123 Cheomdangwagi-ro, Buk-gu, Gwangju, Republic of Korea

## Abstract

**Background:**

The survival of patients with breast cancer is highly sporadic, from a few months to more than 15 years. In recent studies, the gene expression profiling of tumors has been used as a promising means of predicting prognosis factors.

**Methods:**

In this study, we used gene expression datasets of tumors to identify prognostic factors in breast cancer. We conducted log-rank tests and used unsupervised clustering methods to find reciprocally expressed gene sets associated with worse survival rates. Prognosis prediction scores were determined as the ratio of gene expressions.

**Results:**

As a result, four prognosis prediction gene set modules were constructed. The four prognostic gene sets predicted worse survival rates in three independent gene expression data sets. In addition, we found that cancer patient with poor prognosis, i.e., triple-negative cancer, HER2-enriched, *TP53* mutated and high-graded patients had higher prognosis prediction scores than those with other types of breast cancer.

**Conclusions:**

In conclusion, based on a gene expression analysis, we suggest that our well-defined scoring method of the prediction of survival outcome may be useful for developing prognostic factors in breast cancer.

**Electronic supplementary material:**

The online version of this article (doi:10.1186/s12911-016-0292-5) contains supplementary material, which is available to authorized users.

## Background

Breast cancer is one of the most common cancer types in women. In 2015, an estimated 234,190 new cases will be diagnosed, and 40,730 deaths from breast cancer will occur [1]. Prognosis and therapy selection for those with breast cancer are usually affected by clinical and pathology features based on conventional histology and immunohistochemistry findings [2]. In general cases, the menopausal status of the patient, the stage of the disease, the grade of the primary tumor, the estrogen (ER) and progesterone receptor (PR) status, and the level of human epidermal growth factor type 2 receptor (HER2) expression have been used for prognosis predictions. More recently, various uses of molecular profiling in breast cancer also includes ER and PR status testing, HER2/neu receptor status testing, and gene profile testing with, for example, MammaPrint [3] or Ocnotype DX [4, 5].

With regard to clinical intervention, it is critical to identify which patients are at risk of developing a more fatal type of breast cancer. Well-known prognostic factors such as ER and HER2 can be used to predict which patients face higher levels of risk. However, in addition to these traditional makers, there are still novel prognostic factors which are required for predictions of survival for patients with ill-defined breast cancer types. Triple-negative breast cancer is one of the subtypes currently having no such prognostic factors and no targeted drug therapies. Recently, several gene signatures have been identified to predict prognostic outcomes. Tang et al. found that a decreased level of *BECEN1* gene expression in human breast cancer is associated with poor prognosis [6]. The *CENPA* gene was a significantly independent prognostic marker for patients with ER-positive breast cancer [7]. More recently, AI-Ejeh et al. identified eight genes (*MAPT, MYB, MELK, MCM10, CENPA, EXO1, TTK* and *KIF2C*) associated with poor survival in breast cancer patients through biological evidence pertaining to TNBC, metastases, and patient survival [8]. In the latest studies, Liu et al. identified and validated five genes (*CDK1, DLGAP5, MELK, NUSAP1,* and *RRM2*), the expression levels of which were strongly associated with shorten survival time [9]. Although these significant genes were identified, still remains a need for a more comprehensive and exhaustive analysis to find novel prognostic factors.

**Fig. 1:**
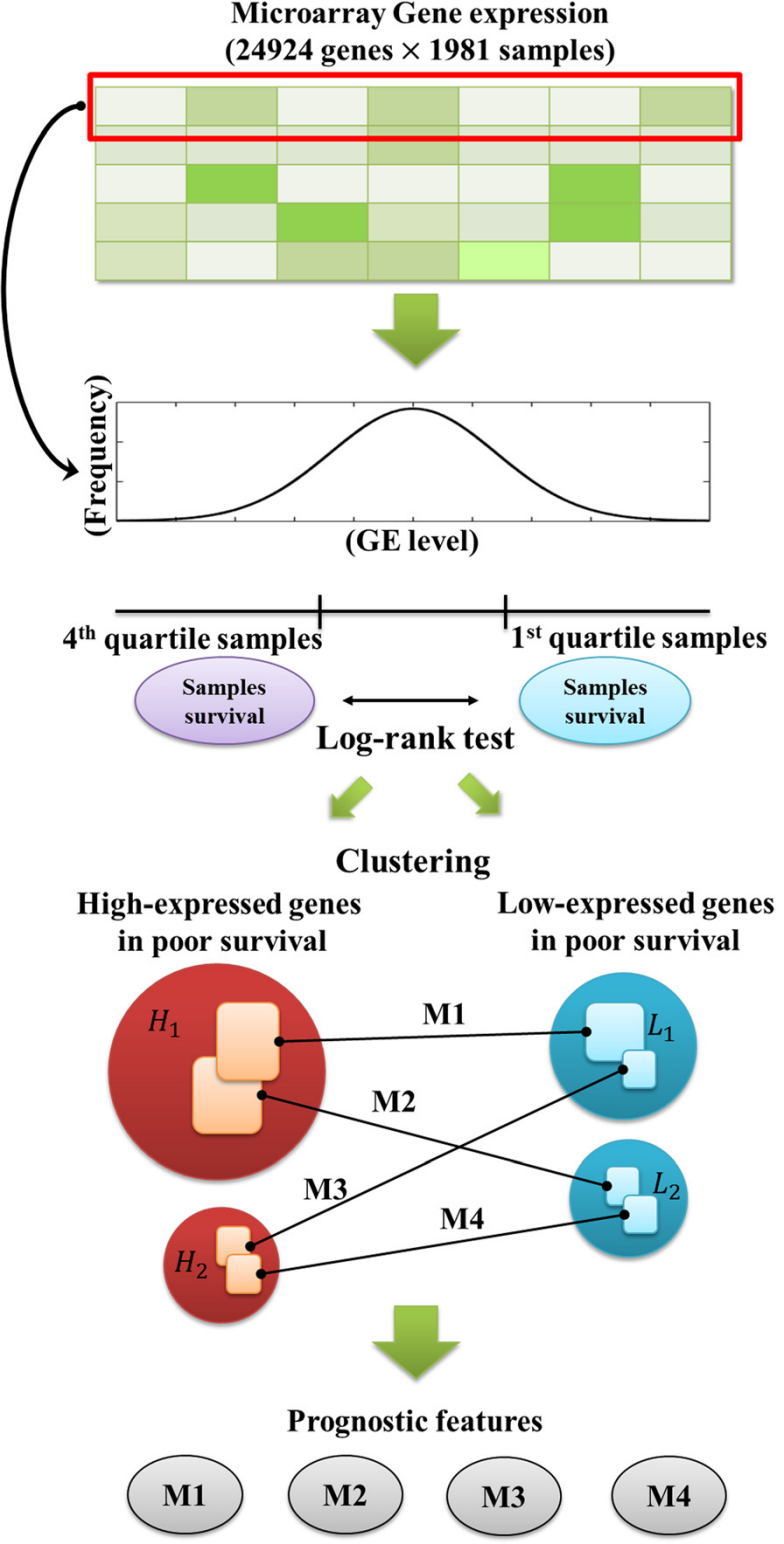
Overall workflow The expression profiles of 24,924 genes were used for the survival log-rank test. Two gene groups were chosen as highly expressed genes (H1, H2), and low-expressed genes (L1, L2) associated with poor survival were clustered according to positive correlations. Negative correlations were used for pairing across differentially expressed gene sets. Finally, four paired gene sets were selected. To estimate the prognostic scores, the ratio of the level of high- and low-expressed genes was defined as the score

In this study, to identify prognostic factors based on gene expressions, we undertook a statistical gene expression data analysis using 1981 breast tumor expression profiles. All of the genes were used individually in our analysis. The expression of each gene was identified as high or low with regard to poor survival, and we clustered genes using an unsupervised method. Finally, we found four matched gene sets along with four modules identified through each gene set which could be used as prognostic markers (Fig. 1). Our results showed that four gene set modules were significantly associated with the worst survival rates; they were strongly associated with a higher tumor grade, *TP53* mutation, ER-negative, HER2-enriched or basal-like subtypes, as well as triple-negative breast cancer.

## Method

### Gene expression profiles

We obtained four independent publicly available breast cancer datasets for survival analysis. One is the Molecular Taxonomy of Breast Cancer International Consortium (METABRIC) in the United Kingdom and Canada [10]. These data were accessed through Synapse (synapse.sagebase.org, Synapse ID: syn1688369). The other three datasets were collected from the Gene Expression Omnibus (GSE25066 [11], GSE2034 [12], GSE3494 [13]). The METABRIC data set is used for training, and three GSE datasets (GSE25066, GSE2034, and GSE3494) are used for validation. To investigate genes related to triple-negative cancer, we used three breast cancer gene expression datasets: METABRIC, GSE2109 [14] and GSE25066. In the METABRIC dataset, normalized expression levels of a total of 54,675 probes were measured in 1981 breast tumor samples. Data were transformed to a compatible 24,924-gene format by selecting the median values of each probe expression. In the three independent GEO datasets, the normalized expression levels of 22,283 probes were measured in 508, 286, and 251 breast tumor samples. These data were also transformed to a 13,433-gene expression format by selecting the median value of each probe expression. Table 1 indicates used five microarray datasets in this study.

**Table 1:** The dataset used in this study

Dataset	grade			age			ER status		TNBC	Total	Platform
	1	2	3	<40	40 ~ 60	>60	+	-			
METABRIC	170	775	952	118	754	1109	1505	435	317	1981	Illumina HT 12v3
GSE25066	32	180	259	85	327	96	297	205	178	508	Affymetrix HG U133A
GSE2034	*NA*	*NA*	*NA*	*NA*	*NA*	*NA*	209	77	*NA*	286	Affymetrix HG U133A
GSE3494	67	128	54	16	90	145	213	34	*NA*	251	Affymetrix HG U133A
GSE2109	31	113	136	*NA*	*NA*	*NA*	*NA*	*NA*	47	351	Affymetrix HG U133A

Here, 1981, 508, 286 and 251 samples of gene expression profiles were used from the METABRIC, GSE25066, GSE22034, and GSE3494 datasets, respectively. The METABRIC data set is used for training, and three GSE (GSE25066, GSE2034, and GSE3494) datasets are used for validation. METABRIC, GSE25066, and GSE2109 datasets were used to find differentially expressed genes (DEGs) between TNBC vs. non-TNBC. The numbers located in table represent the number of samples according to breast cancer characteristics

### Prognostic factor gene set selection

A total of 24,924 genes in METABRIC dataset were used in this research. To identify high/low expressed genes based on patient’s poor survival, we implemented a log-rank test and used an expression fold-change between patients who separated to first quartile and forth quartile corresponding to each gene expression level. This process was implemented by each gene. Hazard ratio was calculated between first and forth quartile patient groups and adjusted *p*-value cutoff was determined as 0.001. Therefore, if hazard ratio is greater than one with proper threshold and patients’ expression fold-change (first/fourth) is greater than 2, we selected the gene as a high-expressed gene in poor survival. Similarly, if hazard ratio is less than one with proper *p*-value cutoff and an expression fold-change (fourth/first) is less than 0.5, we selected the gene as a low-expressed gene in poor survival (Additional file 1: Figure S1). In the log-rank test of every 24,924 gene, we found 413 highly expressed genes associated with poor survival and 411 low-expressed genes associated with poor survival.

### Identification of four prognostic modules

To construct the list of candidate genes for predicting patient’s outcome, we initially used over 20,000 genes and we selected a list of prognostic candidate genes by using a survival log-rank test. However, since too many number of genes showed significance in the log-rank test, we proposed an algorithm for minimizing and clustering genes according to their significance and co-expressed pattern. For clustering the two previously defined gene sets, we used the maximal clique algorithm [15] with Pearson correlation coefficient scores. Among the 413 high-expressed genes, we connected genes if two genes had a Pearson correlation coefficient which exceeded 0.4. We then determined the maximal clique in the 413 genes, after which we eliminated these genes and found the next maximal clique. Similarly, for the 411 low-expressed genes associated with poor survival, we also clustered genes with a minimum Pearson correlation coefficient of 0.4. To avoid the cluster which has too small number of genes, we used only two major clusters. Here, we used clusters for high/low expression gene sets which have over 15 independent genes. After clustering, we obtained two high-expressed gene groups associated with poor survival and two low-expressed gene groups. The connections between the high- and the low-expressed genes were also identified with a Pearson correlation coefficient of -0.4 through the maximal bi-clique generation algorithm [16]. Finally, there were four matched gene sets which are strongly connected to each other, as represented by high correlation values from the gene expression data. Each gene set has high-expressed and low-expressed genes associated with poor survival. Thus, we identified four prognosis prediction scores as the ratio between the median of the high-expressed gene level to the low-expressed gene level in the four matched gene sets. We defined the module 1 score as the ratio of the 26 high-expressed genes associated with poor survival to the 17 low-expressed genes associated with poor survival. Similarly, Modules 2, 3 and 4 scores were respectively defined as the ratios between the eight, nine, and four high-expressed genes associated with poor survival to the 10, nine, and eight low-expressed genes associated with poor survival. Because we used the maximal clique algorithm to cluster each gene set, there was a strong correlation between the expression levels of each high-expressed gene and low-expressed gene associated with poor survival (Pearson’s *r* > 0.4). Between high-expressed genes and low-expressed genes, the maximum Pearson correlation coefficient was found to be -0.4.

### Survival analysis

We analyzed three sets of detailed clinical data from each of the studies used. These were GSE2034, GSE25066, and GSE3494. We used the Disease-Free Survival (DFS) clinical information in GSE2034 and GSE25066, and the Distant Recurrence Free Survival (DRFS) in GSE3494. In a Kaplan Meier survival plots, the median of a measured module’s score was used to dichotomize the data, allowing stratification into high and low groups within each of the three individual datasets.

### Genes associated with triple negative breast cancer

To investigate genes related to triple negative breast cancer (TNBC), after comparing the three independent expression profiles, we selected 230 up-regulated genes and 237 down-regulated genes in TNBC (Cut off *p*-value < 0.05, FDR < 0.05, from *t*-test, log fold change < 0.5) from METABRIC, GSE2109 and GSE25066 datasets.

## Results

### Worse survival with four modules

**Fig. 2:**
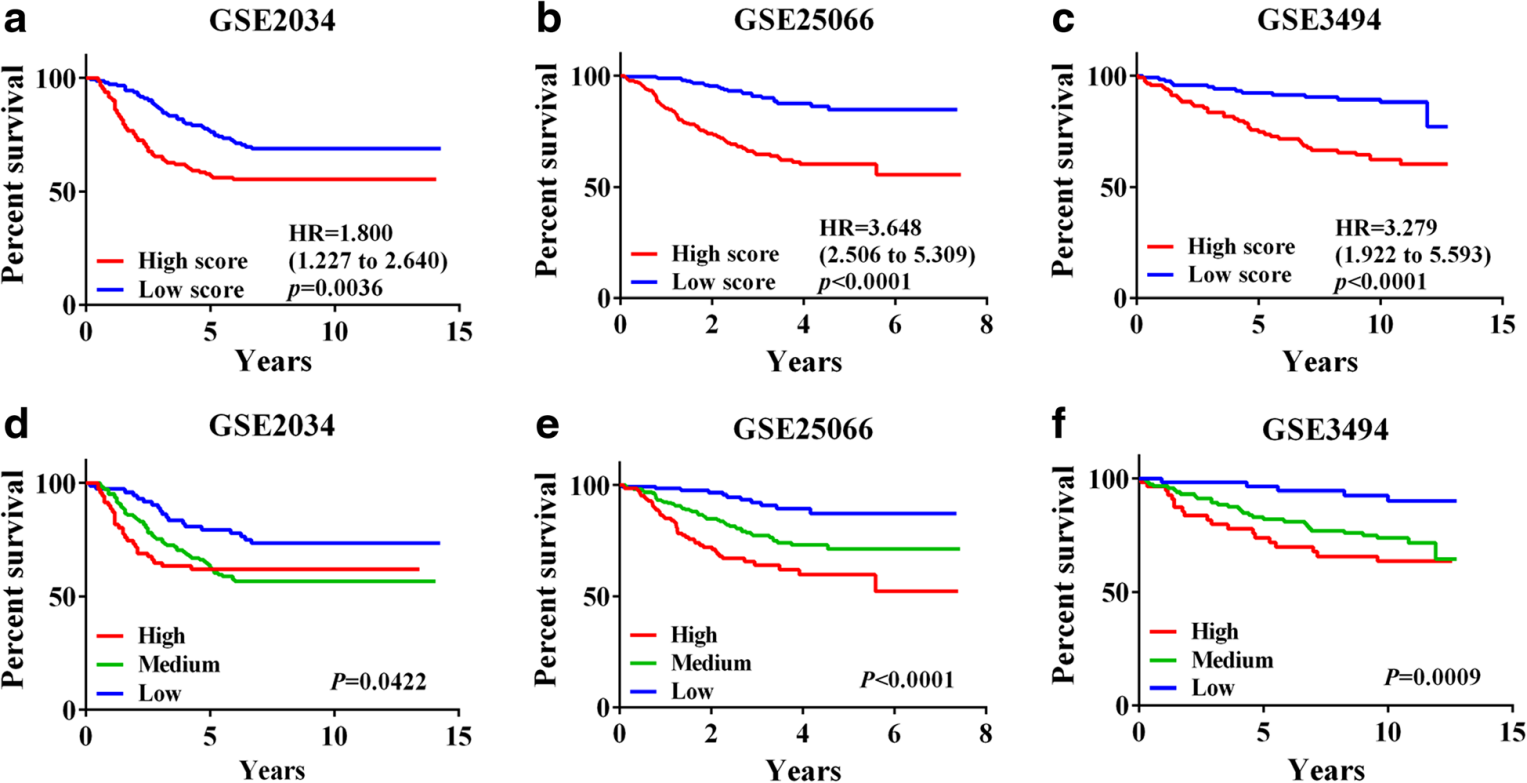
Kaplan-Meier survival plots in the three GEO datasets **a**-**c**. Kaplan-Meier survival plots formulated with module 1 type on GSE25066, GSE2034, and GSE3494. Patients were grouped according to their module scores based on gene expression levels, with scores of over 50 % and less than 50 % representing the high and low scoring groups, respectively. **d**-**f** Kaplan-Meier survival result using module 1. Patients were grouped by module 1 scores based on gene expression levels, with scores of over 25 %, 25–75 %, and 75–100 % representing the high, medium, and low groups, respectively

Patients whose tumors had the highest score among the four modules had the worst prognosis. To validate each score, we used the three datasets of GSE2034, GSE25066, and GSE3494. We selected four matched gene sets from METABRIC data set which consist of about 20,000 genes on Illumina HT 12v3 platform. However, in the test datasets, the expression profiles consist of about 12,000 genes on Affymetrix HG U133A platform. Therefore, all genes obtained from METABRIC were not matched in test datasets. In module 1, among 44 METABRIC genes, 37 genes were used for validation. In module 2, module3 and module 4, we used 11, 27 and 8 matched genes among 17, 36, 14 METABRIC genes, respectively. According to Kaplan Meier survival plots of the three independent sets, high-scoring patients had poor survival rates in the scores of all of the modules. In the GSE2034, GSE25066 and GSE3494 datasets with module 1, patients dichotomized by the prognosis prediction score from 23 high-expressed and 14 low-expressed gene expressions were associated with the worst survival prognosis (Fig. 2a-f). Patients whose tumors had high scores on module 1 had the worst prognosis (*P* = 0.0036, *P* < 0.0001, and *P* < 0.0001, respectively). With modules 2, 3, and 4, similarly, patients whose tumors had high scores had the worst prognosis. In GSE2034, patients whose tumors had high module 1, 2 and 3 scores had the worst. In GSE3494, patients whose tumors had high module 1, 2 and 3 score had the worst prognosis. Only the cases of GSE2034 and GSE3494 with module 4 were not significantly different (Additional file 1: Table S1). We also investigated a lot of possible cases of prediction for differently matched gene set according to manifold threshold. The significance of those results was represented in Additional file 1: Table S1.

### Prognostic factor gene set in module 1

**Fig. 3:**
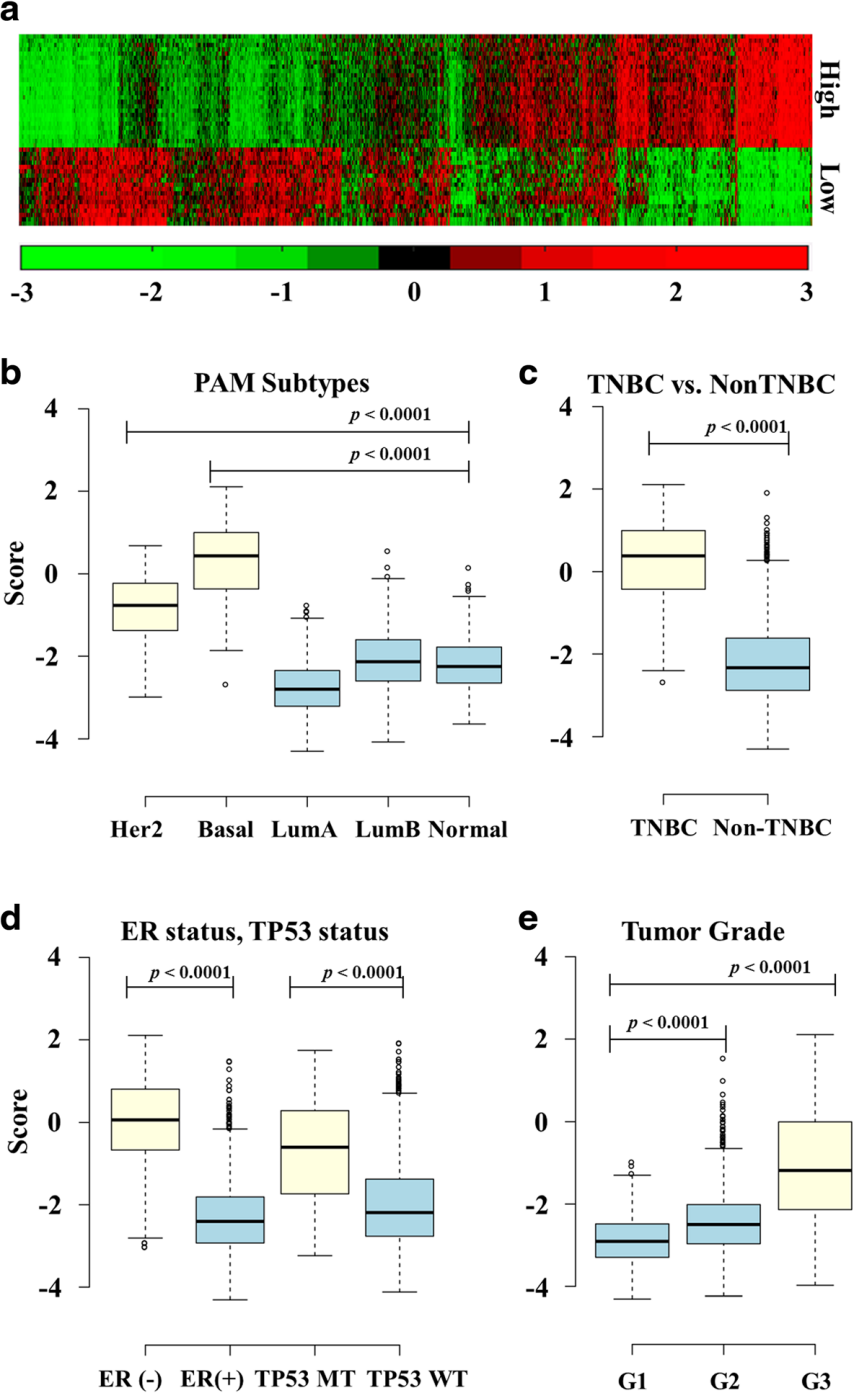
Expression of high/low-expressed genes in poor survival and Boxplots of prognostic score according to breast cancer characteristics. **a** The expression levels of the genes used in module 1. The 26 genes in the upper part of the figure represent high-expressed genes associated with poor survival; and the 17 genes in the lower part represent row-expressed genes associated with poor survival. **b** Boxplot showing the distribution of module 1 scores in the METABRIC dataset according to PAM50 subtypes. **c** Boxplot showing the distribution of module 1 scores according to TNBC and Non TNBC. **d** Boxplot showing the distribution of module 1 scores according to ER and *TP53* mutation status. **e** Boxplot showing the distribution of module 1 scores according to the tumor grade. Units for the ratio between high- to low-expressed genes associated with poor survival (see Methods)

Among the four modules in this research, the module 1 signature strongly represented a high hazard ratio with significant *p*-values (Fig 2a-c). Figure 3a shows the expression pattern of the 28 and 17 prognostic factor genes. The 28 genes in the upper part of the figure represent high-expressed genes associated with poor survival, and these genes are co-expressed relative to each other (Pearson’s *r* > 0.4). The 17 genes in the lower part of the figure represent low-expressed genes associated with poor survival; these genes are also strongly co-expressed relative to each other (Pearson’s *r* > 0.4). In METABRIC, when gene expressions were compared across different tumor types without dichotomization, the module 1 gene score was significantly higher in the HER2-enriched group with the basal-like type (Fig. 3b). Similarly, the triple-negative breast cancer type, ER-negative, and the *TP53* mutation type had higher scores than the other breast cancer types (Fig. 3c and d). The significance levels of the genes used in module 1 were also confirmed using BreastMark [17], which identifies putative prognostic biomarkers. BreastMark gave hazard ratios which were statistically significant for 41 out of 44 genes including previously defined prognostic genes (Table 2, Additional file 1: Table S2). In 26 high-expressed genes in module 1, 16 genes are previously defined as prognostic genes, and among 17 low-expressed genes, 8 genes are previously defined as prognostic genes (Additional file 1: Table S2). 26 genes out of module 1 were differentially expressed genes (DEGs) in TNBC and non-TNBC (19 upregulated genes and 11 down-regulated genes in TNBC) (Table 2).

**Table 2:** The gene list used for module 1

	Gene	Description	METABRIC		BreastMark[17]	
			HR	*p*-value	HR	*p*-value
High-expressed genes	CHEK1 ^a^, ^b^	checkpoint kinase 1	2.16	8.1E-11	1.32	3.9E-06
	FOXM1 ^b^	forkhead box M1	2.58	4.4E-16	1.58	5.5E-13
	CCNA2 ^a^, ^b^	cyclin A2	2.53	5.8E-15	1.47	3.0E-09
	CDC20 ^a^, ^b^	cell division cycle 20	2.50	9.6E-15	1.54	5.8E-13
	TTK ^a^, ^b^	TTK protein kinase	2.28	1.5E-12	1.50	1.2E-11
	CENPA ^a^, ^b^	centromere protein A	2.56	4.4E-16	1.54	6.6E-13
	KIF2C ^a^, ^b^	kinesin family member 2C	2.49	5.6E-15	1.64	2.2E-16
	BUB1 ^a^	BUB1, mitotic checkpoint serine/threonine kinase	2.50	2.1E-14	1.61	2.2E-15
	MCM6	minichromosome maintenance complex component 6	2.09	3.5E-10	1.56	8.8E-14
	LMNB2 ^b^	lamin B2	2.17	3.2E-11	1.38	2.6E-07
	CDC45 ^b^	cell division cycle 45	2.53	2.6E-14	1.50	4.3E-12
	ANLN ^a^	anillin actin binding protein	2.27	6.1E-12	1.48	1.1E-07
	MCM10 ^b^	minichromosome maintenance 10 replication initiation factor	2.30	1.5E-12	1.62	9.8E-14
	CDCA8 ^a^, ^b^	cell division cycle associated 8	2.28	1.0E-12	1.55	3.8E-13
	MELK ^b^	maternal embryonic leucine zipper kinase	2.56	3.6E-15	1.60	0
	CCNB2 ^a^	cyclin B2	2.79	0	1.72	0
	CEP55 ^a^, ^b^	centrosomal protein 55 kDa	2.55	9.1E-15	1.56	1.8E-13
	DLGAP5 ^a^, ^b^	discs, large (Drosophila) homolog-associated protein 5	2.16	3.8E-10	1.46	3.6E-10
	HJURP ^b^	Holliday junction recognition protein	2.79	0	1.61	2.3E-15
	CDCA5 ^a^	cell division cycle associated 5	2.76	0	1.29	1.3E-03
	TRIP13 ^a^, ^b^	thyroid hormone receptor interactor 13	2.18	5.0E-11	1.44	6.6E-09
	GTSE1 ^a^, ^b^	G2 and S-phase expressed 1	2.54	1.7E-14	1.35	5.5E-07
	CDCA3 ^a^, ^b^	cell division cycle associated 3	2.29	5.3E-12	1.48	8.3E-10
	PRR11	proline rich 11	2.09	1.3E-10	1.18	2.6E-06
	FAM83D ^a^	family with sequence similarity 83 member D	2.66	2.2E-16	1.45	2.6E-06
	GTPBP4 ^b^	GTP binding protein 4	1.73	1.6E-06	1.36	4.2E-07
Low-expressed genes	ESR1 ^b^	estrogen receptor 1	0.54	2.6E-08	0.84	2.1E-02
	GATA3 ^b^	GATA binding protein 3	0.57	4.0E-07	0.92	1.6E-01
	LRIG1	leucine-rich repeats and immunoglobulin-like domains 1	0.49	3.0E-10	0.65	1.4E-12
	RABEP1 ^b^	rabaptin, RAB GTPase binding effector protein 1	0.57	5.4E-07	0.75	2.3E-06
	CIRBP ^b^	cold inducible RNA binding protein	0.44	1.1E-12	0.70	3.9E-09
	EVL ^b^	Enah/Vasp-like	0.55	1.1E-07	0.78	1.0E-04
	WDR19	WD repeat domain 19	0.52	1.5E-08	0.77	4.5E-05
	SCUBE2 ^b^	signal peptide, CUB domain, EGF-like 2	0.55	1.7E-07	0.75	1.1E-04
	KIF13B ^b^	kinesin family member 13B	0.55	3.8E-07	0.64	2.1E-11
	TBC1D9 ^b^	TBC1 domain family member 9	0.55	1.2E-07	0.82	9.5E-04
	ANKRA2 ^b^	ankyrin repeat family A member 2	0.55	6.2E-08	0.93	2.3E-01
	DYNLRB2	dynein, light chain, roadblock-type 2	0.49	1.3E-09	0.93	3.9E-01
	NME5 ^b^	NME/NM23 family member 5	0.44	3.8E-12	0.77	4.6E-05
	CAPN8	calpain 8	0.54	1.5E-07	0.67	2.5E-02
	CASC1 ^b^	cancer susceptibility candidate 1	0.44	1.8E-12	0.79	1.0E-04
	BBOF1	basal body orientation factor 1	0.46	2.1E-11	0.78	7.5E-05
	RUNDC1	RUN domain containing 1	0.55	1.0E-07	0.75	3.0E-04

High expressed genes: high-expressed gene group associated with poor survival, Low expressed genes: low-expressed gene group associated with poor survival, ^a^genes associated with the cell cycle process. ^b^Differentially expressed genes between triple-negative and non-triple-negative breast cancer, HR: hazard ratio, *p*-value: log-rank test

## Discussion

The discovery of prognostic factors is crucial work in breast cancer biomarker research. In this study, using a large-scale transcriptomic dataset, we found that four types of prognostic gene sets are strongly related with poor patient outcomes. We used each of the four gene set expressions to evaluate three independent breast tumors and found that scores based on gene expression gave generally consistent predictions of outcomes. When comparing tumor characteristics and scores, tumors with high scores were more likely to have *TP53* mutations, to be HER2-enriched or to have basal-like intrinsic subtypes, triple-negative status, and worse survival rates.

The twenty six genes and 17 genes used in module 1 were strongly co-expressed in METABRIC dataset, and the ratio of the expression levels of the two DEG groups were used as a prognostic marker in this research. Among these high-expressed genes associated with poor survival of patients, many were associated with genes involved in the cell cycle process [18], including several well-defined genes as prognostic factor. Recently, Abdel-Fatah et al. showed that high *CHEK1* expression level is linked to poor prognosis in breast cancer and aggressive breast cancer [19]. *HJURP* was also recently identified as an independent biomarker of cancer outcome in luminal A patients [20]. Breast cancer progression can include the *FOXM1*-*CDCA8* signature which assists as a promising therapeutic target and potential prognostic factor [21]. In addition, Kwok et al. showed that the knockdown of *FOXM1* with thiostrepton in micelle nanoparticles reduced tumor growth rates and increased apoptosis [22]. Thus, they showed that *FOXM1* is one of the primary cellular targets of thiostrepton in breast cancer cells. Karra et al. discovered that high *CDC20* and securin immunoexpression are correlated with unusually poor outcomes of breast cancer patients [23]. *BUB1* has important roles in the proliferation or progression of breast cancer, and the nuclear *BUB1* immunohistochemical status is considered to be an influential prognostic factor in human breast cancer patients [24]. Liu et al. identified and validated five hub genes (*CDK1*, *DLGAP5*, *MELK*, *NUSAP1*, and *RRM2*), the expression levels of which were strongly associated with poor survival. Highly expressed *MELK* revealed poor survival in luminal A/B molecular subtypes of breast cancer [9]. Furthermore, among low-expressed genes associated with poor survival, several well-defined genes were found to be prognostic factor. The role of *GATA3* in breast cancer as a tumor suppressor has been established. Interestingly, the *GATA3* down-regulation is required for the progestin-induced upregulation of cyclin A2(*CCNA2*) and for progestin-induced in vivo and in vitro breast cancer cell growth [25]. Thompsons et al. presented low expression of *LRIG1* is a prognostic factor for breast cancer patients [26]. Cheng et al. showed patients with negative *SCUBE2* protein-expression tumors had worse prognosis than those with positive *SCUBE2* protein-expression tumors in breast cancer [27]. The latest studies suggested the deregulation of *NME5*, *DNALI1* in malignant breast cancer [28]. In addition, 30 genes out of 44 module 1 genes were DEGs in TNBC and non-TNBC (19 upregulated genes and 11 down-regulated genes in TNBC). Thus, we confirmed that the DEGs of classical poor prognosis breast cancer type were also related to our results.

## Conclusions

In conclusion, our finding presents the score of prognosis prediction modules that are strongly associated with shortened survival times in breast cancer, and the score of the module is consistently high in aggressive breast cancer types such as TNBC and ER-negative and HER2-enriched types. In addition, we found that this score is associated with the tumor grade in breast cancer. Thus, we suggest the inclusion of these enriched genes as aggressive cancer markers; 26 co-expressed and 17 genes can be used as new prognostic markers, and we expect that these investigations can be adapted to research on target therapies for ill-defined breast cancer types.

## Ethics approval and consent to participate

Research ethics approval was obtained from the Gwangju Institute of Science & Technology. The METABRIC study protocol was also approved by the ethics committees in previous study [10]. All datasets which were used in this study were composed of anonymized patient information.

## Consent for publication

Not applicable.

## Availability of data and materials

The Molecular Taxonomy of Breast Cancer International Consortium (METABRIC) datasets supporting the conclusions of this article are available in the Synapse repository (https://www.synapse.org/#, Synapse ID: syn1688369). The other four datasets are also available in the Gene Expression Omnibus (http://www.ncbi.nlm.nih.gov/geo/, GSE25066, GSE2034, GSE3494, GSE2109).

## Additional file

Additional file 1:
**Figure S1.** Selection of prognostic candidate-genes based on log-rank test. **Table S1.** The prediction of patients’ outcome based on log-rank test according to varied correlation thresholds. **Table S2.** The gene list of module 1 including previously defined prognostic factor. (DOCX 179 kb)
