# Supplementary material for: Prognostic factor analysis for breast cancer using gene expression profiles
Source: BMC Med Inform Decis Mak. 2016 Jul 18;16(Suppl 1):56. doi: 10.1186/s12911-016-0292-5 (PMC4959370; doi:10.1186/s12911-016-0292-5)
Supplement: Additional file 1: — Figure S1. Selection of prognostic candidate-genes based on log-rank test. Table S1. The prediction of patients’ outcome based on log-rank test according to varied correlation thresholds. Table S2. The gene list of module 1 including previously defined prognostic factor. (DOCX 179 kb) [file 12911_2016_292_MOESM1_ESM.docx]

**Prognostic factor analysis for breast cancer**

**using gene expression profiles**

**Soobok Joe, Hojung Nam**^§^

School of Information and Communication Department, Gwangju Institute of Science and Technology, 123 Cheomdangwagi-ro, Buk-gu, Gwangju, Republic of Korea

^§^Corresponding author

Email addresses:

Soobok Joe: soobok@gist.ac.kr

Hojung Nam: [hjnam@gist.ac.kr](mailto:hjnam@gist.ac.kr)


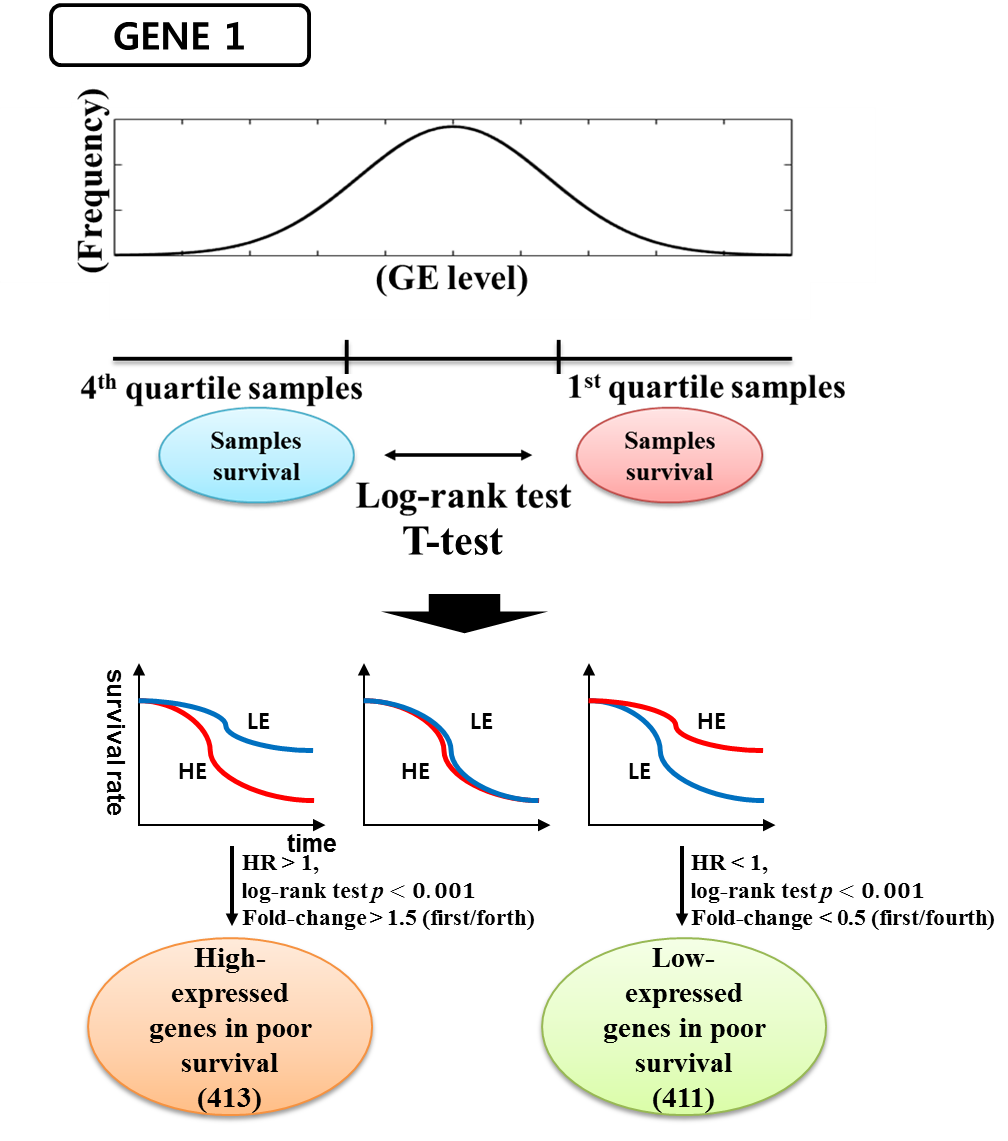


Additional file 1: Figure S1. Selection of prognostic candidate-genes based on log-rank test

To identify high/low expressed genes based on patient’s poor survival, we implemented a log-rank test and used an expression fold-change value of patient groups in the first quartile and forth quartile of the gene expression distribution. This process was implemented per each gene. Hazard ratio was calculated between first and forth quartile patient groups and adjusted *p*-value cutoff was determined as 0.001. Therefore, if hazard ratio is significant and patients’ expression fold-change value (first/forth) is greater than 1.5, we selected the gene as a high-expressed gene in poor survival. Similarly, if hazard ratio is significant and an expression fold-change (first/forth) is less than 0.5, we selected the gene as a low-expressed gene in poor survival.

Additional File 1: Table S1. The prediction of patients’ outcome based on log-rank test according to varied correlation thresholds.

| **Matched**  **Cluster** | | **Correlation (Pearson)** | | **GSE2034** | | **GSE25066** | | **GSE3494** | |
| --- | --- | --- | --- | --- | --- | --- | --- | --- | --- |
|  |  | **Positive** | **Negative** | **HR** | **P** | **HR** | **P** | **HR** | **P** |
| 1 | 1 | 0.4 | -0.4 | **1.800** | **0.004** | **3.648** | **0.000** | **3.279** | **0.000** |
| 1 | 1 | 0.4 | -0.5 | **1.800** | **0.004** | **2.766** | **0.000** | **2.704** | **0.000** |
| 1 | 1 | 0.4 | -0.6 | **1.853** | **0.002** | **2.856** | **0.000** | **3.039** | **0.000** |
| 1 | 1 | 0.5 | -0.4 | 1.488 | 0.052 | **3.572** | **0.000** | **3.642** | **0.000** |
| 1 | 1 | 0.5 | -0.5 | **1.546** | **0.033** | **2.563** | **0.000** | **2.165** | **0.007** |
| 1 | 1 | 0.6 | -0.4 | 1.222 | 0.351 | **3.738** | **0.000** | **1.940** | **0.022** |
| 1 | 1 | 0.6 | -0.5 | **1.852** | **0.002** | **2.368** | **0.000** | **2.729** | **0.000** |
| 1 | 1 | 0.7 | -0.4 | 1.353 | 0.145 | **1.491** | **0.045** | **1.953** | **0.020** |
| 1 | 1 | 0.7 | -0.5 | **1.551** | **0.031** | **1.550** | **0.027** | **2.646** | **0.001** |
| 1 | 2 | 0.4 | -0.4 | **1.544** | **0.033** | **4.005** | **0.000** | **2.368** | **0.002** |
| 1 | 2 | 0.5 | -0.4 | **1.503** | **0.047** | **3.580** | **0.000** | **3.025** | **0.000** |
| 1 | 2 | 0.6 | -0.4 | 1.220 | 0.354 | **4.060** | **0.000** | **1.867** | **0.031** |
| 1 | 2 | 0.6 | -0.5 | 1.160 | 0.506 | **4.070** | **0.000** | **2.128** | **0.008** |
| 1 | 2 | 0.7 | -0.4 | **1.491** | **0.051** | **1.550** | **0.027** | **2.127** | **0.008** |
| 1 | 2 | 0.7 | -0.5 | 1.322 | 0.181 | **1.516** | **0.037** | **2.511** | **0.001** |
| 2 | 1 | 0.4 | -0.4 | **1.525** | **0.039** | **3.218** | **0.000** | **2.665** | **0.001** |
| 2 | 1 | 0.4 | -0.5 | 1.224 | 0.347 | **3.040** | **0.000** | 1.729 | 0.060 |
| 2 | 1 | 0.5 | -0.4 | 1.312 | 0.195 | **3.423** | **0.000** | **2.083** | **0.010** |
| 2 | 1 | 0.5 | -0.5 | 1.415 | 0.092 | **2.889** | **0.000** | **2.133** | **0.008** |
| 2 | 1 | 0.6 | -0.4 | 1.452 | 0.069 | **3.642** | **0.000** | **1.938** | **0.021** |
| 2 | 1 | 0.6 | -0.5 | **1.764** | **0.005** | **2.028** | **0.000** | **2.519** | **0.001** |
| 2 | 1 | 0.7 | -0.4 | **1.414** | **0.092** | **1.768** | **0.004** | **2.755** | **0.000** |
| 2 | 1 | 0.7 | -0.5 | 1.337 | 0.162 | **1.879** | **0.001** | **2.049** | **0.012** |
| 2 | 2 | 0.4 | -0.4 | 1.079 | 0.770 | **3.208** | **0.000** | 1.276 | 0.446 |
| 2 | 2 | 0.4 | -0.5 | 0.946 | 0.851 | **3.774** | **0.000** | 0.971 | 0.977 |
| 2 | 2 | 0.4 | -0.6 | 0.946 | 0.851 | **3.774** | **0.000** | 0.971 | 0.977 |
| 2 | 2 | 0.5 | -0.4 | 1.201 | 0.400 | **3.422** | **0.000** | **2.504** | **0.001** |
| 2 | 2 | 0.5 | -0.5 | 1.325 | 0.178 | **3.099** | **0.000** | 1.549 | 0.139 |
| 2 | 2 | 0.5 | -0.6 | 1.333 | 0.168 | **2.975** | **0.000** | **1.844** | **0.034** |
| 2 | 2 | 0.6 | -0.4 | 1.280 | 0.242 | **3.253** | **0.000** | **2.205** | **0.006** |
| 2 | 2 | 0.7 | -0.4 | **1.627** | **0.016** | 1.330 | 0.161 | **2.133** | **0.008** |

Two major gene clusters are constructed using maximal clique algorithm with Pearson correlation 0.4 to 0.7 as an edge threshold. These processes were implemented on 413 high-expressed genes and 411 low-expressed genes from log-rank test. For each positive correlation threshold, maximal bi-clique algorithm was used with negative edge threshold -0.4 to -0.6.

Additional file 1: Table S2. The gene list of module 1 including previously defined prognostic factor.

|  | **Gene** | **Authors** |
| --- | --- | --- |
| **High- expressed genes** | CHEK1 | Sarah A Andres *et. al* [1] |
|  | FOXM1 | D. C. JIAO *et. al* [2] |
|  | CCNA2 | Tian Gao *et. al* [3] |
|  | CDC20 | H Karra *et. al* [4] |
|  | TTK | Al-Ejeh F *et. al* [5] |
|  | CENPA | Cheng Zhang *et. al* [6]*,* Ashish B. Rajput *et. al* [7] |
|  | KIF2C | Al Muktafi Sadi *et. al* [8] |
|  | BUB1 | Abhik Mukherjee *et. al* [9] *,*Libero Santarpia *et. al* [10] |
|  | MCM6 | None |
|  | LMNB2 | None |
|  | CDC45 | None |
|  | ANLN | Al Muktafi Sadi *et. al* [8] |
|  | MCM10 | None |
|  | CDCA8 | Jiao DC *et. al* [2] |
|  | MELK | Rong Liu *et. al* [11] |
|  | CCNB2 | Emman Shubbar *et. al* [12] |
|  | CEP55 | Katherine J. Martin *et. al* [13] |
|  | DLGAP5 | Rong Liu *et. al* [11] |
|  | HJURP | - Rocío Montes de Oca *et. al* [14] *,* Zhi Hu *et. al* [15] |
|  | CDCA5 | None |
|  | TRIP13 | None |
|  | GTSE1 | None |
|  | CDCA3 | None |
|  | PRR11 | None |
|  | FAM83D | Peter J. Walian *et. al* [16] |
|  | GTPBP4 | None |
| **Low- expressed genes** | ESR1 | Aleksandra Markiewicz *et. al* [17]*,*Sarah A Andres *et. al* [18] |
|  | GATA3 | Franco Izzo *et. al* [19]*,*Nam K. Yoon *et. al* [20] |
|  | LRIG1 | Patricia A. Thompson *et. al* [21] |
|  | RABEP1 | Sarah A Andres *et. al* [18] |
|  | CIRBP | None |
|  | EVL | Sarah A Andres *et. al* [18] |
|  | WDR19 | None |
|  | SCUBE2 | 1. [Chien-Jui Cheng](http://cancerres.aacrjournals.org/search?author1=Chien-Jui+Cheng&sortspec=date&submit=Submit) *et. al* [22] |
|  | KIF13B | None |
|  | TBC1D9 | Sarah A Andres *et. al* [18] |
|  | ANKRA2 | None |
|  | DYNLRB2 | None |
|  | NME5 | Toshima Z. Parris et. al [23] |
|  | CAPN8 | None |
|  | CASC1 | None |
|  | BBOF1 | None |
|  | RUNDC1 | None |

In 26 high-expressed genes in module 1, 16 genes are previously defined as prognostic genes, and among 17 low-expressed genes, 8 genes are previously defined as prognostic genes. These genes are listed through the PubMed (http://www.ncbi. nlm.nih.gov/pubmed) search in terms of (GENE[TIAB] AND (breast cancer[TIAB] OR breast tumor[TIAB])) AND (prognosis [TIAB] OR prognostic[TIAB]) .

**References**

1. Abdel-Fatah TM, Middleton FK, Arora A, Agarwal D, Chen T, Moseley PM, Perry C, Doherty R, Chan S, Green AR *et al*: **Untangling the ATR-CHEK1 network for prognostication, prediction and therapeutic target validation in breast cancer**. *Molecular oncology* 2015, **9**(3):569-585.

2. Jiao D, Lu Z, Qiao J, Yan M, Cui S, Liu Z: **Expression of CDCA8 correlates closely with FOXM1 in breast cancer: public microarray data analysis and immunohistochemical study**. *Neoplasma* 2014, **62**(3):464-469.

3. Gao T, Han Y, Yu L, Ao S, Li Z, Ji J: **CCNA2 is a prognostic biomarker for ER+ breast cancer and tamoxifen resistance**. *PloS one* 2014, **9**(3):e91771.

4. Karra H, Repo H, Ahonen I, Löyttyniemi E, Pitkänen R, Lintunen M, Kuopio T, Söderström M, Kronqvist P: **Cdc20 and securin overexpression predict short-term breast cancer survival**. *British journal of cancer* 2014, **110**(12):2905-2913.

5. Al-Ejeh F, Simpson PT, Sanus JM, Klein K, Kalimutho M, Shi W, Miranda M, Kutasovic J, Raghavendra A, Madore J *et al*: **Meta-analysis of the global gene expression profile of triple-negative breast cancer identifies genes for the prognostication and treatment of aggressive breast cancer**. *Oncogenesis* 2014, **3**:e100.

6. Zhang C, Han Y, Huang H, Min L, Qu L, Shou C: **Integrated analysis of expression profiling data identifies three genes in correlation with poor prognosis of triple-negative breast cancer**. *International journal of oncology* 2014, **44**(6):2025-2033.

7. Rajput AB, Hu N, Varma S, Chen CH, Ding K, Park PC, Chapman JA, Sengupta SK, Madarnas Y, Elliott BE *et al*: **Immunohistochemical Assessment of Expression of Centromere Protein-A (CENPA) in Human Invasive Breast Cancer**. *Cancers* 2011, **3**(4):4212-4227.

8. Wang D-Y, Youngson BJ, Miller N, Boerner S, Done SJ, Leong WL: **Clinical relevance of DNA microarray analyses using archival formalin-fixed paraffin-embedded breast cancer specimens**. *BMC cancer* 2011, **11**(1):253.

9. Mukherjee A, Joseph C, Craze M, Chrysanthou E, Ellis IO: **The role of BUB and CDC proteins in low-grade breast cancers**. *The Lancet* 2015, **385**:S72.

10. Santarpia L, Iwamoto T, Di Leo A, Hayashi N, Bottai G, Stampfer M, André F, Turner NC, Symmans WF, Hortobágyi GN: **DNA repair gene patterns as prognostic and predictive factors in molecular breast cancer subtypes**. *The oncologist* 2013, **18**(10):1063-1073.

11. Liu R, Guo C-X, Zhou H-H: **Network-based approach to identify prognostic biomarkers for estrogen receptor–positive breast cancer treatment with tamoxifen**. *Cancer biology & therapy* 2015, **16**(2):317-324.

12. Shubbar E, Kovács A, Hajizadeh S, Parris TZ, Nemes S, Gunnarsdóttir K, Einbeigi Z, Karlsson P, Helou K: **Elevated cyclin B2 expression in invasive breast carcinoma is associated with unfavorable clinical outcome**. *BMC cancer* 2013, **13**(1):1.

13. Martin KJ, Patrick DR, Bissell MJ, Fournier MV: **Prognostic breast cancer signature identified from 3D culture model accurately predicts clinical outcome across independent datasets**. *PLoS One* 2008, **3**(8):e2994.

14. de Oca RM, Gurard-Levin ZA, Berger F, Rehman H, Martel E, Corpet A, de Koning L, Vassias I, Wilson LO, Meseure D: **The histone chaperone HJURP is a new independent prognostic marker for luminal A breast carcinoma**. *Molecular oncology* 2015, **9**(3):657-674.

15. Hu Z, Huang G, Sadanandam A, Gu S, Lenburg ME, Pai M, Bayani N, Blakely EA, Gray JW, Mao J-H: **The expression level of HJURP has an independent prognostic impact and predicts the sensitivity to radiotherapy in breast cancer**. *Breast Cancer Res* 2010, **12**(2):R18.

16. Walian P, Hang B, Mao J: **Prognostic significance of FAM83D gene expression across human cancer types**. *Oncotarget* 2015.

17. Markiewicz A, Wełnicka-Jaśkiewicz M, Skokowski J, Jaśkiewicz J, Szade J, Jassem J, Żaczek AJ: **Prognostic significance of ESR1 amplification and ESR1 PvuII, CYP2C19* 2, UGT2B15* 2 polymorphisms in breast cancer patients**. *PloS one* 2013, **8**(8).

18. Andres SA, Brock GN, Wittliff JL: **Interrogating differences in expression of targeted gene sets to predict breast cancer outcome**. *BMC cancer* 2013, **13**(1):326.

19. Izzo F, Mercogliano F, Venturutti L, Tkach M, Inurrigarro G, Schillaci R, Cerchietti L, Elizalde PV, Proietti CJ: **Progesterone receptor activation downregulates GATA3 by transcriptional repression and increased protein turnover promoting breast tumor growth**. *Breast Cancer Research* 2014, **16**(6):491.

20. Yoon NK, Maresh EL, Shen D, Elshimali Y, Apple S, Horvath S, Mah V, Bose S, Chia D, Chang HR: **Higher levels of GATA3 predict better survival in women with breast cancer**. *Human pathology* 2010, **41**(12):1794-1801.

21. Thompson PA, Ljuslinder I, Tsavachidis S, Brewster A, Sahin A, Hedman H, Henriksson R, Bondy ML, Melin BS: **Loss of LRIG1 locus increases risk of early and late relapse of stage I/II breast cancer**. *Cancer research* 2014, **74**(11):2928-2935.

22. Cheng C-J, Lin Y-C, Tsai M-T, Chen C-S, Hsieh M-C, Chen C-L, Yang R-B: **SCUBE2 suppresses breast tumor cell proliferation and confers a favorable prognosis in invasive breast cancer**. *Cancer research* 2009, **69**(8):3634-3641.

23. Parris TZ, Kovács A, Aziz L, Hajizadeh S, Nemes S, Semaan M, Forssell‐Aronsson E, Karlsson P, Helou K: **Additive effect of the AZGP1, PIP, S100A8 and UBE2C molecular biomarkers improves outcome prediction in breast carcinoma**. *International Journal of Cancer* 2014, **134**(7):1617-1629.
